# Mitochondrial Entry of Cytotoxic Proteases: A New Insight into the Granzyme B Cell Death Pathway

**DOI:** 10.1155/2019/9165214

**Published:** 2019-05-21

**Authors:** Denis Martinvalet

**Affiliations:** Department of Biomedical Science, University of Padova and Veneto Institute of Molecular Medicine, Via G. Orus 2, 35129 Padova, Italy

## Abstract

The mitochondria represent an integration and amplification hub for various death pathways including that mediated by granzyme B (GB), a granule enzyme expressed by cytotoxic lymphocytes. GB activates the proapoptotic B cell CLL/lymphoma 2 (Bcl-2) family member BH3-interacting domain death agonist (BID) to switch on the intrinsic mitochondrial death pathway, leading to Bcl-2-associated X protein (Bax)/Bcl-2 homologous antagonist/killer- (Bak-) dependent mitochondrial outer membrane permeabilization (MOMP), the dissipation of mitochondrial transmembrane potential (ΔΨm), and the production of reactive oxygen species (ROS). GB can also induce mitochondrial damage in the absence of BID, Bax, and Bak, critical for MOMP, indicating that GB targets the mitochondria in other ways. Interestingly, granzyme A (GA), GB, and caspase 3 can all directly target the mitochondrial respiratory chain complex I for ROS-dependent cell death. Studies of ROS biogenesis have revealed that GB must enter the mitochondria for ROS production, making the mitochondrial entry of cytotoxic proteases (MECP) an unexpected critical step in the granzyme death pathway. MECP requires an intact ΔΨm and is mediated though Sam50 and Tim22 channels in a mtHSP70-dependent manner. Preventing MECP severely compromises GB cytotoxicity. In this review, we provide a brief overview of the canonical mitochondrial death pathway in order to put into perspective this new insight into the GB action on the mitochondria to trigger ROS-dependent cell death.

## 1. Introduction

Cytotoxic T lymphocytes (CTL) and natural killer (NK) cells are essential to the host defense against pathogen-infected or transformed cells [1–6]. They trigger target cell death either through the death receptor pathway or through the cytotoxic granule pathway, which relies on perforin-dependent delivery of granzyme serine proteases into the cytosol of the target cell [7–19]. In humans, 5 granzymes (A, B, H, K, and M) have been identified, whereas mice have 10 orthologs (A, B, C, D, E, F, K, L, M, and N) [20–22]. Granzyme B (GB) and granzyme A (GA) are the most abundantly expressed and consequently the best characterized [20–22]. GA cleaves its substrates after lysine or arginine residues to trigger a caspase-independent, B cell CLL/lymphoma 2- (Bcl2-) insensitive, and mitochondrial outer membrane permeabilization- (MOMP-) independent cell death pathway with the morphological feature of apoptosis [23–27]. GB cleaves its substrates after aspartic acid residues to induce cell death either in a caspase-dependent or caspase-independent manner [22, 28–31]. Human GB can also directly cleave key effector caspase substrates, such as the inhibitor of caspase-activated DNAses (ICAD), the DNA damage sensor poly(ADP-ribose) polymerase (PARP-1), the nuclear structural protein lamin, the nuclear mitotic apparatus protein (NuMa), the DNA-dependent protein kinase catalytic subunit (DNA-PK_c_), and the microtubule protein tubulin, to activate death similar to that induced by the caspase pathway [26, 29, 32–36].

The mitochondria represent an integration and amplification hub for various death pathways including that of GB. Similarly, to initiator caspases, GB activates the proapoptotic Bcl-2 member BID to switch on the intrinsic mitochondrial death pathway [34–37]. This leads to dissipation of mitochondrial transmembrane potential (ΔΨm) and Bax- and Bak-dependent MOMP. MOMP is necessary for the release of apoptogenic factor cytochrome c (cyt c), HtrA2/Omi, endonuclease G (Endo G), Smac/Diablo, and apoptosis-inducing factor (AIF) from the mitochondrial intermembrane space to the cytosol [26, 38–42]. Interestingly, human GB can also induce loss of ΔΨm and cyt c release in the presence of caspase inhibitors, and mice deficient for BID, Bax, and Bak, critical for MOMP, are still sensitive to GB-induced cell death, indicating that human GB targets the mitochondria in other ways; this will be discussed in greater detail later [38, 40, 43, 44]. Much emphasis has been put on MOMP, as it is an important step in the mitochondrial death pathway. However, the contribution of other mitochondrial alterations such as reactive oxygen species (ROS) production for the GB cell death pathway and apoptosis in general has received less attention. Interestingly, GA, GB, and caspase 3 are all able to directly target the mitochondrial respiratory chain complex I for ROS-dependent cell death. Research focusing on the ROS biogenesis in this pathway has revealed that GB must enter the mitochondria for ROS production, making the mitochondrial entry of cytotoxic proteases (MECP) an unexpected critical step in the granzyme death pathway. For general review on the granzymes, we refer the readers to PMID: 18304003, 12360212, and 22095283.

## 2. Reactive Oxygen Species

Nowadays, it is accepted that ROS production is a determinant of many cell death mechanisms, including apoptosis, necrosis/necroptosis, ferroptosis, pyroptosis, and autophagic cell death [45–52]. ROS are also involved in the physiology and pathophysiology of many processes and conditions such as signal transduction, ischemia/reperfusion, stroke, neurodegenerative disorders, aging, and cancer [53–58]. ROS are formed by the partial reduction of oxygen. They encompass both radical species, which have unpaired electrons, e.g., superoxide anion (O^2-·^), hydroxyl radical (^·^OH), and nitric oxide (NO), and nonradical products, which do not have unpaired electrons but are powerful oxidizing agents, e.g., hydrogen peroxide (H_2_O_2_), hypochlorous acid (HOCl), and peroxynitrite (ONOO^−^) [59]. The primary radical species (O^2-·^, NO, and H_2_O_2_) are produced by specialized enzyme systems such as the nicotinamide adenine dinucleotide phosphate hydrogen (NADPH) oxidases, the myeloperoxidases, the nitric oxide synthases (NOS), the monooxygenase activity of cytochrome P450, the xanthine oxidase, the monoamine oxidase (MAO), and the mitochondrial respiratory chain, with the latter being the most prominent source of endogenous ROS [59, 60]. Counterintuitively, ROS are also necessary for physiological functions. Indeed, because the primary radical species can easily be controlled by enzymatic and nonenzymatic antioxidants such as superoxide dismutase, catalase, and glutathione, and because their reactions with biomolecules are reversible, they are particularly capable of physiological/pathophysiological intracellular signaling. Actually, primary radical species are continuously generated through several physiological processes in the cell and are crucial for inflammation, vasoconstriction, signal transduction, and cell migration, differentiation, and proliferation [57, 58, 61–68].

Nevertheless, excessive ROS production has deleterious effects on cells. Although, even at high concentrations, the primary species (O^2-·^, NO, and H_2_O_2_) are not directly damaging to the cells, they react with themselves or with metal ions to produce the deleterious highly reactive secondary species ^·^OH, ONOO^−^, and HOCl [69]. A well-known example is the Fenton reaction of H_2_O_2_ with iron ions to produce ^·^OH [69]. These secondary species are highly toxic and poorly controlled and react irreversibly with almost all classes of biomolecules, resulting in oxidative damage and cellular dysfunction [70–74]. Overproduction of such secondary species leads to a state of oxidative stress in which the endogenous antioxidant machinery of the cell is overwhelmed. Consequently, the cells accumulate damage within macromolecules like DNA, lipids, and proteins [70–74]. To cope with the deleterious potential of the secondary radical species, cells evolved a robust antioxidant machinery based on both enzymatic and nonenzymatic antioxidants, such as superoxide dismutase (SOD), catalase, glutathione, and thioredoxin systems. SOD occurs in three isoforms: cytosolic CuZn-SOD (SOD1), mitochondrial Mn-SOD (SOD2), and extracellular EC-SOD (SOD3) [56, 75]. SOD, as its name indicates, dismutes O^2-^ into H_2_O_2_ [75]. Catalase is a homotetramer that converts H_2_O_2_ into water in the presence of NADPH [56, 76]. The glutathione peroxidases (GPx), in association with glutathione (GHS), reduce H_2_O_2_ and lipid hydroperoxides [56, 77]. There are eight GPxs, all tetrameric enzymes with the particularity of using selenocysteine in their active sites (GPx1-4 and GPx6), while GPx5 and GPx7-8 are nonselenium congeners [77]. Moreover, the removal of H_2_O_2_ also involves thioredoxin (TRX), thioredoxin reductase (TRR), thioredoxin peroxidase (PRX), and glutaredoxins [56]. Most of these enzymatic antioxidants use NADPH as a reducing equivalent. NADPH not only maintains catalase in the active form but also functions as a cofactor of TRX and glutathione reductase for the recycling of oxidized glutathione (GSSG) to its reduced form (GSH), for later use as a cosubstrate by GPx [56, 76, 77]. The most abundant nonenzymatic antioxidant in the cell is GSH, which participates in the reduction of H_2_O_2_ into H_2_O and O_2_, and is thereby oxidized to form GSSG. GSSG is then reduced into GSH by glutathione reductase using NAD(P)H as an electron donor. It maintains ascorbic acid (vitamin C) and *α*-tocopherol (vitamin E) in their active forms. GSH also protects from cell death by interfering with proapoptotic and antiapoptotic signaling cascades. Vitamin C and E are, respectively, aqueous and lipophilic antioxidants that protect the intra- and extracellular milieu and membranes from oxidants. As stated earlier, when the cellular antioxidant machinery is overrun, cells accumulate damage that can be fatal. Initially, ROS were considered by-products of cell death. However, new evidence suggests that ROS have a major role in the initiation and amplification of the death insult by modulating many signaling pathways. Although they are contributing determinants for various forms of cell death, their biogenesis and their mode of action during cell death are still not well understood except for ferroptosis.

## 3. Apoptosis

Apoptosis is orchestrated via a genetically encoded molecular machinery dedicated to cell death. This programmed cell death is necessary for the normal development and homeostasis of multicellular organisms. Therefore, any dysregulation of this sophisticated machinery contributes to the etiology of a vast spectrum of pathologies, including cancer and neurodegenerative disorders [39, 51, 78]. We refer the readers to PMID: 20683470, 25236395, and 17237344 for reviews on cell death. Morphologically, cells undergoing apoptosis shrink and assume a round shape as a result of the caspases protease-mediated degradation of cytoskeleton proteins. This is followed by condensation of the chromatin into compact patches against the nuclear envelope (pyknosis), disruption of the nuclear envelope, and fragmentation of DNA (karyorrhexis). The cell membrane shows irregular buds known as blebs [51, 78–80]. Ultimately, the cells break apart into several vesicles called apoptotic bodies, which are then phagocytized. *In vivo* cells committed to apoptosis are phagocytized before the end of this process, avoiding collateral damage and inflammation. Consequently, apoptosis is in general seen as nonimmunogenic [51, 78–80]. However, in certain conditions, this process can become immunogenic [81]. Two pathways lead to apoptosis: the extrinsic pathway, which is initiated by the engagement of death receptors at the cell surface [80, 82–85], and the intrinsic pathway, which is triggered downstream of cellular stress such as DNA damage, endoplasmic reticulum (ER) stress, or growth factor withdrawal [80, 82, 86]. In both pathways, the mitochondria play a critical role either by amplifying or by engaging the death insult, respectively. These two pathways crosstalk with the activation of the executioner caspase.

## 4. The Extrinsic Pathway

The extrinsic pathway is engaged after stimulation of the death receptors, tumor necrosis factor receptor (TNFR), FAS, and TNF-related apoptosis-inducing ligand receptor (TRAILR) at the cell surface by their respective ligands TNF, FASL, and TRAIL [80, 82–84]. The ligand binding results in trimerization of the receptors and recruitment of adaptor molecules such as FAS-associated death domain protein (FADD) and then procaspase 8 to form the death induction signaling complex (DISC) through homotypic interaction of their death domain (DD) or death effector domain (DED). As a consequence, dimerization occurs along with proximity induced activation of the initiator caspase 8, which can then directly cleave and activate caspase 3 and caspase 7 for the execution of apoptosis [85, 87]. Interestingly, caspase 8 can also proteolytically activate BID, connecting the extrinsic pathway with the intrinsic pathway. Caspase 8 cleaves and activates BID into its truncated form (tBID), which activates BAX and BAK for MOMP [80, 82–84].

## 5. The Intrinsic Pathway

As stated earlier, mitochondria are central to the execution of apoptosis. MOMP is considered the point of no return. Indeed, in stressed conditions, proteins of the Bcl2 family member with only the BH3 domain (the BH3-only proteins) [Bcl2-interacting mediator of cell death/Bcl-2-like protein 11 (BIM/Bcl2-L-11), Bcl2-associated agonist of cell death (BAD), Bcl-2-interacting killer (BIK), Bcl-2-modifying factor (BMF), BCL2/adenovirus E1B 19 kDa protein-interacting protein 3 (BNIP3), activator of apoptosis harakiri (HRK), phorbol-12-myristate-13-acetate-induced protein 1 (PMAIP1/NOXA), and Bcl-2-binding component 3/p53 upregulated modulator of apoptosis (PUMA)] transduce the death signals that originate from stressed conditions for activation of the proapoptotic BAX and BAK [51, 88–90]. This results in conformational changes, leading to BAX and BAK oligomerization at the mitochondrial outer membrane (MOM) for MOMP. This succession of events has drastic consequences for cell fate, as it leads to the release of the apoptogenic factor cyt c from the mitochondrial intermembrane space to the cytosol. Cytosolic cyt c is required for the oligomerization of the adaptor protein apoptotic protease-activating factor 1 (APAF1) and the formation of the apoptosome, a scaffold dedicated to the proteolytic conversion of the initiator procaspase 9 into active caspase 9. Active caspase 9 processes and activates the executioner and effector caspase 3 and 7 necessary for the orchestration of cellular dismantling that causes cell death. Other apoptogenic factors, such as second mitochondrion-derived activator of caspase/Diablo (Smac/Diablo**)** and high-temperature requirement protein A2 (HtrA2), are also released from the mitochondrial intermembrane space in order to unleash effector caspases from the inhibitory action of the inhibitors of apoptosis (IAPs) such as XIAP [91]. Endo G and AIF are also released from the mitochondria to further nucleosomal DNA fragmentation [41, 42].

## 6. Regulation of Apoptosis

In both the extrinsic and intrinsic pathways, MOMP is tightly regulated by the interplay of the Bcl2 proteins, as the antiapoptotic members, Bcl2, Bcl-X_L_, and MCL1, counterbalance the proapoptotic function of BAX and BAK [92, 93]. Upon death stimuli, activation of the BH3-only proteins will unleash the proapoptotic action of BAX and BAK by antagonizing the antiapoptotic members [92, 93]. Post MOMP, caspase activation is also regulated by the IAP proteins IAP1/2 and XIAP, a set of cytosolic factors containing one or more baculovirus IAP repeat motifs necessary for interaction with the caspase. The IAP also contains a RING domain for the recruitment of E_2_ ubiquitin-conjugating enzymes. Upon caspase binding, the IAP mediates their ubiquitination and proteasome-dependent degradation [91–96]. The formation of the apoptosome is dependent on dATP and cyt c, although ATP at a physiological level and transfer RNA inhibit cyt c [97, 98]. This suggests that enough cyt c must be available to overcome this inhibition. Likewise, physiological concentrations of calcium and potassium ions inhibit apoptosome formation in a cyt c-sensitive manner [99, 100]. Lastly, chaperone proteins PHAP/pp32, Hsp70, and Hsp90 favor apoptosome activation by preventing APAF1 aggregation [101–103].

## 7. The Caspases

The caspases are divided into two subfamilies. Caspases 2, 3, 6, 7, 8, 9, and 10 are involved in cell death initiation and execution, while caspases 1, 4, 5, 13, and 14 are dedicated to cytokine processing during inflammatory responses [104–106]. The initiator caspases 2, 8, 9, and 10 have a long prodomain, while the executioner caspases 3, 6, and 7 have a small one. Initiator caspase activation depends on its proximity dimerization after binding to the adaptor protein with the death domain motif. Once activated, they proteolytically activate the executioner caspases. Active caspases are heterotetramers composed of two large and two small subunits [104–106]. The active executioner caspases orchestrate the cleavage of a discrete set of proteins to induce the morphological and biochemical features associated with apoptosis.

## 8. The Mitochondria

It is now accepted that the mitochondria originated from the Rickettsia group of alpha-proteobacteria, eubacteria-like endosymbionts [107, 108]. However, a recent metagenomics analysis suggests that the mitochondria ancestors originated most likely from a proteobacterial lineage that branched off before the divergence of all sampled alpha-proteobacteria [109]. Structurally speaking, the mitochondria are double-membrane organelles made of an outer membrane (MOM) surrounding a highly folded inner membrane (MIM), which protrudes into an inner compartment consisting of the mitochondrial matrix. Although the MIM is separated from the MOM by an intermembrane space (IMS), both mitochondrial membranes remain connected at areas of contact sites, which are involved in the organization of the MIM invagination called cristae [110–113]. In eukaryotic cells, mitochondria have an undisputed role in cellular energy production and metabolism [114–116]. Simply said, the mitochondria are the cellular power house because they are proficient at producing ATP via oxidative phosphorylation (OXPHOS) [117]. The OXPHOS system embedded in the MIM receives reduced electrons from NADH and FADH2 at the level of complex I and complex II, respectively. These electrons tunnel to complex III via coenzyme Q10 and then to complex IV via cyt c and to the final acceptor oxygen to produce water (H_2_O). This electron flow provides energy, which is transiently stored in the form of a proton gradient as it is coupled with the efflux of protons from the matrix to the IMS. The resulting proton-motive force is used to fuel complex V for ATP synthesis [60]. Even in physiological conditions, this electron transport is associated with mitochondrial ROS production at the level of complexes I and III [60]. Furthermore, recent evidence also indicates that dimers of complex V are likely the molecular determinants of the permeability transition pore involved in Ca^2+^-dependent cell death [118–120]. Mitochondria are also crucial for Ca^2+^ homeostasis, cell cycle regulation, differentiation, cell death, and aging [49, 51, 121–127]. This plethora of functions is matched by their morphological and structural versatility. In fact, mitochondria are extremely dynamic interconnected tubular networks constantly undergoing remodeling through fusion and fission events [128, 129]. The mitochondrial shaping proteins, a family of dynamin-related GTPases, and their adaptor proteins orchestrate the balance between fusion and fission. Mitofusin (MFN) 1 and 2 inserted in the MOM and optic atrophy 1 (OPA1) anchored in the MIM control the fusion of the MOM and MIM, respectively [124, 130–135]. Mitochondrial fission requires the translocation of dynamin-related protein (DRP) 1 from the cytosol to the mitochondria where it docks on the MOM to its adaptor human fission protein 1 (hFis1), mitochondrial fission factor (MFF), and mitochondrial dynamics 51 kDa and 49 kDa proteins (MiD51 and MiD49) [124, 130–139]. Interestingly, mitochondria share contact sites with the endoplasmic reticulum (ER) [124, 130–135, 140, 141]. These mitochondrial ER contact sites (MERCs) regulate mitochondrial Ca^2+^ homeostasis, lipid transfer, the initiation of autophagosome formation, and determination of the mitochondrial fission site [142–149]. At the MERCs, defined by the ER tubules wrapping the mitochondria, the mitochondria are constricted [142, 150]. In fact, the MERCs provide a platform for the recruitment of motor force-generating cytoskeletal proteins [150]. ER-bound inverted formin 2 (INF2) concentrates between the two organelles where the ER wraps the mitochondria [146, 150, 151]. INF2 triggers the assembly of the actomyosin motor, which provides the force for the initial constriction of the mitochondria [142, 146, 150, 151]. Once assembled, the ER-associated constricted mitochondria enable polymerized DRP1 to spiral around the mitochondria to mediate their fission [142, 146, 150–154]. Mitochondria can respond to many cellular cues. For example, during starvation, the pool of cellular AMP rises, leading to the activation of protein kinase A (PKA) that phosphorylates DRP1 on serine 637, preventing its translocation to the mitochondria and therefore blocking its profission activity. Consequently, mitochondria elongate because unopposed fusion likely serves as a mechanism to spare these organelles from autophagic degradation in order to optimize energy production in response to starvation conditions [130, 131, 133, 134]. During stress, mitochondrial depolarization triggers an intracellular Ca^2+^ rise that activates the phosphatase calcineurin, which dephosphorylates DRP1 at serine 637, leading to its activation and consequently mitochondrial fragmentation in order to induce cell death [130, 131, 133, 134]. Furthermore, OPA1 also regulates cyt c release by controlling the mitochondrial cristae junctions [133, 155–157]. Accumulating evidence also suggests a direct relationship between mitochondrial fragmentation and apoptosis. During apoptosis, Bax colocalizes with DRP1 and MFN2 at the fission site. Formation of the BID/Bax/Bak complex reduces mitochondrial fusion, probably due to the inhibition of MFN2, while it stabilizes the docking of sumoylated DRP1 on the MOM, leading to mitochondrial fragmentation [158–161]. In this context, mitochondrial fragmentation is caspase independent. During oxidative stress, protein kinase C triggers phosphorylation of human DRP1 isoforms 1 and 3 at residues S616 and S579, respectively, resulting in mitochondrial fragmentation [162]. On the other hand, the loss of the OPA1 long isoforms that results in mitochondrial fragmentation is also observed during cell death [163, 164]. Taken together, these findings indicate that the contribution of mitochondria to cell death is far more complex than originally appreciated.

## 9. Complexity of Cytochrome c Release

The MOMP is necessary for apoptogenic factor release. This permeabilization can result from Bax Bak oligomerization and translocation at the MOM or from membrane rupture due to mitochondrial swelling after a lasting episode of permeability transition pore (PTP) opening [88, 165]. The mitochondrial respiratory chain complexes reside in the cristae membrane along with ATP synthase dimers, with the latter found at the tip of the cristae to maintain their curvature [166], whereas cyt c is found in the cristae space. Interestingly, the narrow cristae junction is maintained by oligomers of a mixture of long and short isoforms of OPA1 [157]. This indicates that in order for the release of cyt c following Bax/Bak-dependent MOMP, this cristae junction must be disrupted. In fact, it was demonstrated that tBID disrupts OPA1 oligomers in order to trigger the cristae junction remodeling necessary for the proper release of cyt c [157]. Moreover, cyt c and Endo G are engaged in electrostatic and hydrophobic interactions with cardiolipins, suggesting that they must be untethered from the membrane for optimal release. Interestingly, ROS disrupt these weak bonds to promote their release upon MOMP [167–169]. In the absence of caspase activity, cells still die following MOMP induction although with a slower pace. This is most likely due to the dissipation of the mitochondrial membrane potential and the release of endonuclease G and AIF. Interestingly, caspase 3 contributes to the loss of mitochondrial potential following the cleavage of NDUFS1, leading to the loss of the respiratory complex I function, which results in a decrease in ATP production and increase in ROS production [50]. However, although the resulting mitochondrial ROS suppress the immunogenicity of HMGB1 by oxidation, they promote cell death by oxidizing the released cyt c. In fact, highly glycolytic cells such as neurons and cancer cells have increased stores of GSH due to the exacerbation of the pentose phosphate pathway (PPP). In such cells, following MOMP, cytosolic cyt c tends to be reduced, rising the threshold for full caspase activation [51, 170].

## 10. New Insight into the Granzyme B Mitochondrial Pathway

**Figure 1:**
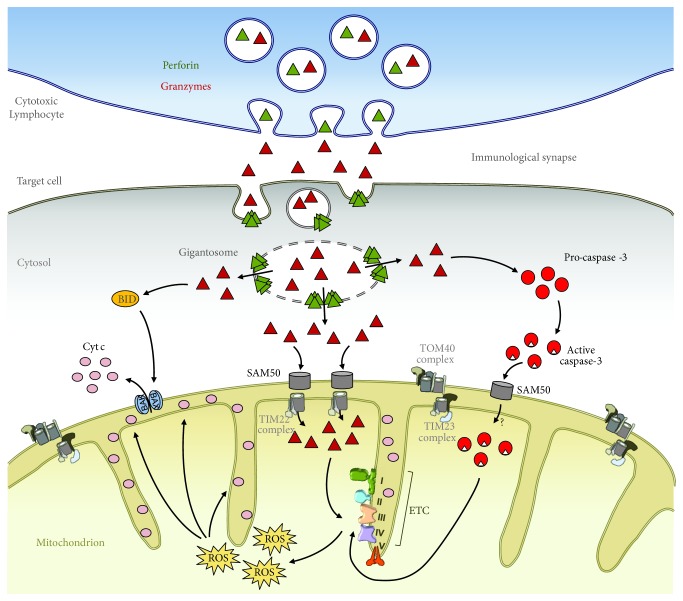
Granzymes and caspase 3 enter the mitochondria through the Sam50 channel. Upon recognition of the target cell, the effector cell vectorially degranulates the cytotoxic granule content into the immunological synapse from where perforin, a pore forming protein, triggers membrane repair, which results in the internalization and delivery of the granzymes into the target cell cytosol. In the target cell cytosol, granzymes initiate cell death by cleaving various substrates. Both granzymes and caspase 3 cross the outer and the inner mitochondrial membrane through the Sam50 and Tim22 channels, respectively. Once in the matrix, granzyme and caspase 3 disrupt the electron transport chain (ETC) complex I and trigger ROS mediation of cell death.

As stated earlier, human GB can directly cleave key caspase substrates, such as BID, ICAD, PARP-1, lamin, NuMa, DNA-PK_c_, and tubulin, to activate the mitochondrial and DNA damage pathways similar to the caspase pathway [20, 32, 33]. The GB mitochondrial pathway leads to ROS production and dissipation of the ΔΨm and MOMP, together with the release of apoptogenic factors such as cyt c, HtrA2/Omi, endonuclease G, Smac/Diablo, and AIF from the mitochondrial IMS to the cytosol [29–31, 35, 36, 38–40]. Human GB also induces loss of ΔΨm and release of cyt c in the presence of caspase inhibitors, and mice deficient for BID, Bax, and Bak, which are critical for MOMP, are still sensitive to GB-induced cell death [38, 40, 43, 44], indicating that human GB can also attack the mitochondria via a different mechanism. Although they activate distinct death pathways, GA and GB have in common the ability to induce cell death in a ROS-dependent manner. In fact, we showed that both GA and GB target the NADH:ubiquinone oxidoreductase complex I of the electron transport chain by cleaving the subunits NDUFS3, NDUFV1, and NDUFS2 [25, 171–173]. Cleavage of complex I subunits leads to a rapid and robust mitocentric ROS production, loss in complex I, II, and III activity, disorganization of the respiratory chain, impaired mitochondrial respiration, and loss of mitochondrial cristae junction [25, 171–174]. Interestingly, caspase 3 acts similarly on complex I by cleaving NDUFS1 to induce ROS-dependent death [50]. Overall, it appears that three different death pathways (GA, GB, and caspase 3) crosstalk at the level of the mitochondrial respiratory chain complex I to induce ROS-dependent death. Although GA, GB, and caspase 3 do not have a mitochondrial targeting sequence, they still penetrate this double-membrane organelle independently of the translocase of the outer membrane (TOM40) and of the inner membrane (TIM23) complexes, which represent the canonical mitochondrial protein import pathway to the matrix. Instead, we found that GA, GB, and caspase 3 cross the MOM through the Tob55/Sam50 channels and the MIM though Tim22 in a mtHSP70-dependent manner [174]. This mitochondrial entry requires an intact mitochondrial membrane potential (Figure 1) [174]. We found that GB lysine 243 (K243) and arginine 244 (R244) were necessary for its mitochondrial translocation. Substitution of these two residues to alanine did not alter GB catalytic activity but was enough to prevent entry of GB into target cell mitochondria upon delivery by killer cells. Interestingly, preventing GB entry into the mitochondria, either by K243A/R244A substitution or by silencing Sam50, severely alters the cytotoxicity of GB [174]. These results clearly indicate that GB must enter the mitochondria in a process we have coined mitochondrial entry of cytotoxic proteases (MECP) for efficient cell death.

The TOM40-TIM23 complexes are involved in mitochondrial biogenesis through their essential role in mitochondrial protein import [175–177]. Conceptually, if we think at the TOM40-TIM23 complexes not only as translocases but also as safe keepers of the mitochondrial integrity because of their selectivity of the imported proteins, the fact that cytotoxic molecules aimed at destroying the mitochondria use Tob55/Sam50-Tim22 as a side door to enter these organelles makes sense (Figure 1). Notably, both Tob55/Sam50 and Tim22 are dedicated to the insertion of proteins in the mitochondrial membrane and were not intended to be used as “translocases” [178]. It is therefore possible that some mechanistic aspect of this common function could be hijacked by cytotoxic molecules. Granzyme mitochondrial entry breaks all the codes of mitochondrial import, something that could be expected from proteins aimed at destroying the mitochondrial functions for irrevocable cell death. Moreover, blocking access of granzyme and caspase 3 to the mitochondria compromises their ability to induce cell death, suggesting that MECP is an unanticipated critical step in ROS-dependent cell death.

In the case of GB, we have clearly shown that MECP is independent of MOMP, since it occurs in Bax and Bak double-knockout cells [88, 171, 174]. Moreover, granzyme and caspase 3 mitochondrial entry is dependent on the mitochondrial membrane potential [171, 174]. The fact that MOMP depolarizes the mitochondria indicates that MECP must take place before MOMP or in mitochondria where MOMP does not occur. Yet we have shown that GB-mediated ROS potentiates apoptogenic factor release. Our results suggest that MOMP, although required, could in fact be the tip of the iceberg. Our data indicate that granzymes A and B and caspase 3 use SAM50 as a channel translocase for MECP, and this translocase activity seems sensitive to SAM50 phosphorylation status, raising the question of how MECP is regulated. Moreover, GA and GB trigger extensive mitochondrial fragmentation that could also be ROS-dependent. We also observed that GB triggers loss of cristae junction in isolated mitochondria [171]. This interesting observation fits well with the rout of GB mitochondrial entry. Indeed, Sam50 interacts with the MICOS complex to maintain the architecture of the mitochondrial cristae [112, 113, 179–181]. It is reported that loss of Mic60 or Mic10 results in a complete loss of cristae junction [112]. Whether upon exiting the Sam50 channel, GB can alter some of the MICOS component for the observed loss of cristae junction needs to be investigated. Moreover, considering the mitochondrial membrane disruption and cristae opening, BID/Bax/Bak-dependent MOMP [35, 36, 88, 133] and its consequence, the actual release of apoptogenic factors as two dependent steps, the hierarchy of molecular events between MECP, ROS production, mitochondrial fragmentation, MOMP, and apoptogenic factor release must be clearly established for GB. In future studies, this hierarchy of events should be investigated in order to understand their interdependence.

The core subunits of mammalian complex I are similar to those of the elementary bacterial complex I [182]. Therefore, it is not surprising that granzyme can also cleave bacterial complex I. As a matter of fact, it was demonstrated that CTL kill intracellular bacteria following bacterial complex I disruption. This requires perforin-mediated granulysin and granzyme delivery into the infected target cell cytosol where granulysin allows granzyme to cross the bacterial cell wall. Once in the bacteria, GA and GB disrupt bacterial complex I subunits and oxidative stress response enzymes such as SOD and catalase [183]. Interestingly, it was also recently reported that CTL eradicate protozoan parasites (*Trypanosoma cruzi*, *Toxoplasma gondii*, and *Leishmania major*) through perforin-mediated granulysin and granzyme delivery into parasites for the cleavage of proteins involved in oxidative defense or oxidoreduction reactions (these parasites do not express a conserved respiratory chain complex I) [184]. These results further underline the significance of ROS production and of targeting complex I or ROS-generating oxidoreductive enzymes for cell death induction, as it has been clearly showed that these two processes are conserved across phylum from bacterial to protozoan and to mammals [171, 173, 183, 184]. GB also induces the death of nonoxidative bacteria by targeting highly conserved sets of proteins involved in the biosynthetic and metabolic pathways that are critical for bacterial survival under diverse environmental conditions [185]. Because mitochondria have a bacterial origin, one can expect granzyme to target similar sets of the biosynthetic and metabolic mitochondrial pathways, as it does in bacteria.

GB-induced mitochondrial ROS are necessary for optimal apoptogenic factor release, rapid DNA fragmentation, and rupture of lysosomal membranes [171, 172]. However, the mechanisms by which ROS contribute to these hallmarks of cell death remain incompletely understood. As stated earlier, cyt c is bound to cardiolipins by both electrostatic and hydrophobic interactions that are destabilized by ROS to enable its optimal release from the mitochondria upon MOMP induction [167, 168]. Similarly, ROS are implicated in the proper release of Endo G from the mitochondria [169]. We found that GB-induced ROS enhanced apoptogenic factor release. The antioxidant NAC inhibited P and GB-mediated cyt c, Endo G, and Smac release from the mitochondria [171]. Overexpression of GB-uncleavable NDUFV1, NDUFS1, and NDUFS2, which reduced GB-mediated ROS production, also inhibited GB-induced apoptogenic factor release; thus, GB induction of mitocentric ROS promotes apoptogenic factor release upon MOMP. Our results indicated that the release of apoptogenic factors requires at least two independent steps—MOMP, which is BID/Bax/Bak-dependent, and MECP, which is essential for the increase in ROS necessary to untether the apoptogenic factors from the cardiolipin to facilitate their release. Another hallmark of GB-mediated cell death is caspase-activated DNAse- (CAD-) mediated oligonucleosomal DNA fragmentation [41, 186]. This oligonucleosomal DNA fragmentation was also reduced by NAC antioxidant treatment and overexpression of GB-uncleavable NDUFV1, NDUFS1, and NDUFS2; thus, ROS production is necessary for GB-mediated apoptotic DNA damage. This could partly be explained by the fact that Endo G, the release of which is ROS-dependent, cooperates with CAD for optimal apoptotic DNA fragmentation. ROS oxidize DNA to form abasic sites [70]. It is possible that such oxidative DNA damage facilitates CAD and Endo G-mediated oligonucleosomal DNA fragmentation. It is also possible that the direct effect of the ROS on the nucleocytoplasmic transport could modulate the subcellular localization of these apoptotic DNAses in order to favor karyorrhexis. However, additional studies are required to test these hypotheses.

We are beginning to understand how ROS contribute to cell death, and a full understanding of the molecular mechanism(s) by which ROS regulate cell death will require characterization of the molecular targets of ROS. Whether ROS-dependent death requires nonspecific oxidation of various macromolecules or of a discrete subset of ROS targets still needs to be established. Moreover, characterization of the most effective radical species requires further investigation. It is likely that secondary radical species play critical roles. Furthermore, the amounts of ROS needed for irrevocable cell death induction remain unknown. Lastly, whether ROS from dying cells can signal to neighboring cells and the role of such putative paracrine signaling also need to be investigated.

## 11. Conclusion

The mitochondria serve as a hub for the integration and amplification of multiple death pathways including that of GB. We found that, in addition to the canonical BID/Bax/Bak-dependent MOMP, GB must enter the mitochondria to be fully cytotoxic. Mitochondrial entry of GB requires residues K243 and R244 and is mediated though the Sam50 channel. This new discovery suggests that MECP is an unanticipated novel step in the mitochondrial death pathway. Our results also suggest that the five human granzymes accumulate in the mitochondria, and this was clearly demonstrated to be Sam50-dependent for at least GA, GB, and GM. Finally, our findings indicate that MECP is also necessary for some actions of caspase 3 in mitochondria. In the future, it will be interesting to test whether other cytotoxic proteases follow the same path to the heart of the mitochondria to determine the extent to which MECP is conserved among other cell death pathways.
